# BEAGLE 3: Improved Performance, Scaling, and Usability for a High-Performance Computing Library for Statistical Phylogenetics

**DOI:** 10.1093/sysbio/syz020

**Published:** 2019-04-23

**Authors:** Daniel L Ayres, Michael P Cummings, Guy Baele, Aaron E Darling, Paul O Lewis, David L Swofford, John P Huelsenbeck, Philippe Lemey, Andrew Rambaut, Marc A Suchard

**Affiliations:** 1 Center for Bioinformatics and Computational Biology, University of Maryland, College Park, MD 20742, USA; 2 Department of Microbiology, Immunology and Transplantation, Rega Institute, KU Leuven – University of Leuven, 3000 Leuven, Belgium; 3 The ithree Institute, University of Technology Sydney, Ultimo, New South Wales 2007, Australia; 4 Department of Ecology and Evolutionary Biology, University of Connecticut, Unit 3043, Storrs, CT 06269, USA; 5 Florida Museum of Natural History, University of Florida, Gainesville, FL 32611, USA; 6 Department of Integrative Biology, University of California, Berkeley, CA 94720 USA; 7 Institute of Evolutionary Biology, University of Edinburgh, King’s Buildings, Edinburgh EH9 3FL, UK; 8 Fogarty International Center, National Institutes of Health, Bethesda, MD 20892, USA; 9 Department of Biomathematics University of California, Los Angeles, CA 90095, USA; 10 Department of Biostatistics, University of California, Los Angeles, CA 90095, USA; 11 Department of Human Genetics, University of California, Los Angeles, CA 90095, USA

**Keywords:** Bayesian phylogenetics, GPU, maximum likelihood, multicore processing, parallel computing

## Abstract

BEAGLE is a high-performance likelihood-calculation library for phylogenetic inference. The BEAGLE library defines a simple, but flexible, application programming interface (API), and includes a collection of efficient implementations for calculation under a variety of evolutionary models on different hardware devices. The library has been integrated into recent versions of popular phylogenetics software packages including BEAST and MrBayes and has been widely used across a diverse range of evolutionary studies. Here, we present BEAGLE 3 with new parallel implementations, increased performance for challenging data sets, improved scalability, and better usability. We have added new OpenCL and central processing unit-threaded implementations to the library, allowing the effective utilization of a wider range of modern hardware. Further, we have extended the API and library to support concurrent computation of independent partial likelihood arrays, for increased performance of nucleotide-model analyses with greater flexibility of data partitioning. For better scalability and usability, we have improved how phylogenetic software packages use BEAGLE in multi-GPU (graphics processing unit) and cluster environments, and introduced an automated method to select the fastest device given the data set, evolutionary model, and hardware. For application developers who wish to integrate the library, we also have developed an online tutorial. To evaluate the effect of the improvements, we ran a variety of benchmarks on state-of-the-art hardware. For a partitioned exemplar analysis, we observe run-time performance improvements as high as 5.9-fold over our previous GPU implementation. BEAGLE 3 is free, open-source software licensed under the Lesser GPL and available at https://beagle-dev.github.io.

Statistical phylogenetic analyses based on maximum likelihood (ML) and Bayesian inference are computationally challenging because of the intensive nature of the calculations required. At the core of statistical phylogenetics is the calculation of the likelihood (probability) of the observed molecular sequence character states under a specific model of evolution using a recursive algorithm (Felsenstein 1981), and the computation of this calculation comprises most of the running time for analyses. Decreasing the time (wall clock) for this computation is the *raison d’être* for the BEAGLE library, a parallel computing platform for high-performance calculation of phylogenetic likelihoods that makes efficient use of the fine-scale parallelization capabilities of computer processors, especially graphics processing units (GPUs)(Suchard and Rambaut 2009; Ayres et al. 2012). Here, we describe and evaluate important changes made to BEAGLE in the time since we introduced Version 1.0 of the library in this journal in 2012. We also set out general scalability and usability expectations for users.

## Key Improvements

The most significant improvements to the library can be broadly divided into two categories: new implementations that expand the breadth of parallel computing hardware that can be efficiently exploited, and parallel-algorithm advances that improve the GPU implementation for nucleotide-model analyses.

### New Implementations for Broader Hardware Support

BEAGLE offers a single application programming interface (API) backed with a wide range of hardware-specific implementations that aim to provide efficient use of available resources. For GPUs, previous versions of BEAGLE only used the CUDA parallel computing framework. Although this remains the most efficient way to target NVIDIA devices, CUDA is proprietary and incompatible with GPUs from other manufacturers. For the CPU (central processing unit), previous versions of the library have included single-core implementations using vectorization intrinsics (e.g., SSE) to achieve efficient performance (SSE [Streaming SIMD Single Instruction, Multiple Data Extensions] and AVX [Advanced Vector Extensions] are two of several sets of processor intrinsics for vectorizing numeric computation). Additional parallelization across multiple cores required the calling software to partition data into multiple data subsets, each of which is computed in a separate BEAGLE instance executing in separate a thread (threading is a method of achieving concurrent processing within or among processor cores). Although this approach (of CPU threading at the program level) is a natural fit for independently modeled subsets, it is ill-suited for finer-grained parallelization. Moreover, it places the nontrivial task of managing threads on the application developer. BEAGLE 3 adds new GPU and CPU implementations to target a wider range of manycore processors and to facilitate multicore parallelism.

#### OpenCL

Based on our existing CUDA implementation for GPUs (Suchard and Rambaut 2009; Ayres et al. 2012), we have added new GPU and CPU implementations that use the OpenCL framework, an open standard for parallel computing devices. We achieved this by modifying the previous CUDA host-side code to a framework-independent one that is usable for both CUDA or OpenCL implementations. This generic parallel-implementation model communicates with the CUDA and OpenCL APIs through a single internal interface that, in turn, has an implementation available for each framework. Further significant sharing of code between CUDA and OpenCL exists at the device kernel level. There is a single set of kernels for both frameworks, with keywords for each being defined at the preprocessor stage. Although there is a common kernel code-base across frameworks, functions that impart a crucial effect on performance are differentiated for each hardware type. This allows for distinctly optimized parallel implementations: one for CUDA GPUs, one for OpenCL GPUs and one for modern $CDATA[$CDATA[$\times$$86 devices such as multicore CPUs with vectorization extensions. The level of specialization among evolutionary models, processors, and frameworks results in over 1300 distinctly compiled device kernels (Ayres and Cummings 2017b).

#### CPU threading

Despite the open nature and broad industry support for OpenCL, we recognize that it is an external framework that is not always available to users of the library. Thus, we have added a native threading option to our CPU implementation. This allows BEAGLE to harness the increasing capability of modern CPUs for parallel processing, in a more portable manner. Specifically, we have added an implementation for multicore CPUs that uses a pool of C++ threads (C++ Standards Committee and Others 2011) to process independent site patterns concurrently. We found that this approach, using native functionality in the C++ standard, allowed us to add thread-parallelism to our existing, low level, SSE vectorization of character states (Ayres et al. 2012, Ayres and Cummings 2017b) in an efficient and well-performing manner. The number of threads created varies automatically with problem size, up to the core count of the processor. Alternatively, client programs can set a limit on the number of threads.

### Improved GPU Implementation for Nucleotide Analyses

Previously, strong GPU performance (speedup $CDATA[$CDATA[$>2\times$$) for nucleotide-model phylogenetic analyses required data sets that shared the same evolutionary model across many ($CDATA[$CDATA[$>10^{3}$$) unique site patterns. Due to the low number of states each nucleotide character can assume, smaller sequences failed to saturate the large number of cores available on GPUs. This resulted in poor performance for many analyses. In order to increase core utilization and improve GPU performance, we have identified additional opportunities for parallel computation with nucleotide-model analyses and implemented them in BEAGLE 3.

#### Data partitions

Statistical phylogenetic analyses benefit from increases in modeling flexibility. One clear way of improving model flexibility is to allow (conditionally) independent estimation of model parameters for distinct character data subsets (e.g., genes, codon positions). This is typically referred to as a partitioned model and is a technique available in all phylogenetic software packages that currently support BEAGLE. Until Version 3, partitioned analyses with BEAGLE have required the client program to create multiple instances of the library, one for each data subset defined by the partitioning scheme. When BEAGLE instances shared the same GPU, they were executed in sequence, thus incurring significant performance and memory inefficiencies, especially for nucleotide problems with small ($CDATA[$CDATA[$<10^{3}$$ unique site patterns) data subsets.

#### Tree traversal

Another category of analysis that performed inefficiently on GPUs was that of nucleotide data sets with many sequences (tips) but without a large number unique site patterns ($CDATA[$CDATA[$<10^{3}$$). The amount of parallelization afforded by the limited number of unique sites failed to saturate the hardware capacity of GPUs. The GPU parallelization of the phylogenetic likelihood function only acted on a fine scale, exploiting parallelism to accelerate the calculation of partial likelihood arrays at each internal node in the proposed tree, with the traversal of the tree itself occurring in a sequential manner. Thus, problems with few unique site patterns were always *small* for parallel processing purposes and thus not amenable to speedups, independent of tree size.

#### Parallel computation of partial likelihood arrays

What these two previously underperforming categories of nucleotide-model problems, partitioned data sets and large trees with shorter sequence lengths, have in common is that they include many independent partial likelihood arrays that were being computed in series. We have augmented the BEAGLE API (in a backwards-compatible manner) and developed new parallel implementations for CUDA and OpenCL frameworks to identify and execute the concurrent computation of independent partial likelihood arrays.

Our solution for concurrent computation of partial likelihood arrays involves different approaches depending on problem size and hardware type. For nucleotide sequences with more than $CDATA[$CDATA[$10^{3}$$ unique site patterns on NVIDIA devices, we use multiple CUDA streams, directing independent computation to separate streams. The use of CUDA streams also benefits analyses that employ multiple Markov chains, as these can now be more efficiently computed in parallel on a single GPU. For data sets with fewer unique site patterns, on both CUDA and OpenCL, we use newly developed device kernels that can process multiple partial likelihood arrays concurrently in a single execution launch (Ayres and Cummings 2017a).

Although the BEAGLE API remains backwards-compatible, programs that use the library will require adaptation to enable the above improvements. For partition-defined data subset concurrency, independent subsets need to share a library instance. For parallel tree traversal, partial-likelihood operations need to be sent to BEAGLE in a reverse level-order manner (in contrast to the typical postorder approach). Further tree traversal parallelism can be gained by rerooting the proposed tree when appropriate (Ayres and Cummings 2018).

Enabling this further concurrency of computation in BEAGLE 3 allows a wider range of phylogenetic inferences to benefit from parallel computing hardware. Nucleotide-model analyses with many small data subsets or with large trees but few site patterns, can now achieve higher levels of hardware utilization. Additionally, memory usage for partitioned analyses is significantly reduced as the overhead imposed by multiple library instances is eliminated. For data sets with many subsets this improvement can cut memory usage by more than half (see Scalability section).

## Performance Evaluation

Here, we explore the performance effect of the key improvements to the library using a computationally challenging data set. We compare speedups for the previous and current versions of BEAGLE on various parallel hardware resources, using BEAST (Suchard et al. 2018) (v1.10.5) and MrBayes (Ronquist et al. 2012) (v3.2.7), two popular programs for Bayesian statistical phylogenetics.

### Benchmark Setup

For these benchmarks, we examine a dengue virus data set with 997 genomes spanning the global dengue diversity and a total of 6869 unique site patterns across 10 gene-based subsets (data set available in source code repository, see Availability section). Although we use the same data set for both BEAST and MrBayes analyses, some model parameters and prior assumptions differ, so we make no attempt to compare inference across these software packages.

We specifically choose a data set with a large number of sequences and with many independent subsets, each with few unique site patterns, to best showcase the gains in concurrency achieved in this version of the library. Previously, data sets with these characteristics have been the most challenging for effective parallelization. BEAGLE-enabled peak performance for data sets with many more patterns and using higher state-count models are reported in the Scalability section below as well as elsewhere (Ayres et al. 2012; Ayres and Cummings 2017b; Baele et al. 2018). To analyze this data set, we use a nucleotide-model and assume that each subset evolves at a different relative rate, according to an independent HKY substitution model (Hasegawa et al. 1985) and with rate variation among sites in each data subset modeled by a discretized gamma distribution with four rate categories (Yang 1996). For BEAST, we employed a recently developed adaptive multivariate normal transition kernel that allows the concurrent estimation of a large number of parameters, split across partitioned data, by exploiting parallel processing (Baele et al. 2017).

For each inference benchmark, we run a single Markov chain for $CDATA[$CDATA[$10^{5}$$ iterations and use the double-precision floating-point format in the BEAGLE-enabled runs. We assess speedups relative to the double-precision likelihood calculator for each program. For MrBayes, we also show results for the native, AVX + fused multiply-add (FMA) vectorized, implementation in single precision, which is the default when not using BEAGLE. For BEAST, the use of BEAGLE is required, and thus we use the default, non-vectorized, CPU implementation in BEAGLE as the performance baseline. For the BEAST benchmarks we use Version 1.10.5 and for MrBayes we use Version 3.2.7. These versions use the latest API methods in BEAGLE 3 to improve performance.

**Figure 3.**
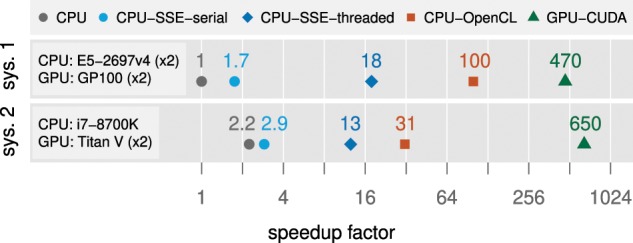
Relative performance gains (fold-speedup) on two systems for analysis with BEAST v1.10.5 and BEAGLE v3.1.2 for an unpartitioned codon analysis of the previously described dengue virus data set (see Performance Evaluation section), comprising 3330 codons. For the BEAGLE GPU implementation, this data set requires 21.5 GB of memory, and thus we scale computation by pattern block across two library instances, each running on a separate GPU. Benchmarks are for 10,000 iterations, and the single-threaded, non-vectorized, version of BEAGLE CPU running on *System 1* is used as a reference.

As a basis for comparison we include results for BEAGLE 2, the previous major release of the library. Version 2 was released in 2014, however, there was no accompanying application note. The main improvement in Version 2 relative to the first release is the addition of an OpenCL implementation. Specifically, we compare Versions 2.1.3 and 3.1.2 of BEAGLE and report results for three implementations of the library, under different hardware resources on two different systems. The CPU-SSE implementation on *System 1* (an HPC [high-performance computing] platform) runs on two Intel Xeon E5-2697v4 processors, with a total of 36 cores. On *System 2* (a high-end desktop machine), it uses an Intel i7-8700K CPU with 6 cores. This implementation uses SSE vectorization, and in BEAGLE 3 it additionally uses CPU threads to parallelize computation. This threading is in addition to that performed by BEAST, which by default employs one thread per partition-defined data subset (MrBayes does not natively support multithreading). We benchmark the CUDA version of BEAGLE on *System 1* using the NVIDIA GP100 GPU. For *System 2,* we test on the current state-of-the-art NVIDIA GPU, the Tesla-generation Titan V. We test the OpenCL implementation on an Advanced Micro Devices (AMD) R9 GPU on *System 1*. On *System 2*, we use the top-of-the-line AMD GPU, the Radeon Vega Frontier. We do not include results for our OpenCL implementation on the CPUs for nucleotide data, as we have found it to consistently underperform the threaded version for nucleotide-model analyses (for codon-model benchmarks using OpenCL on the CPU see Fig. 3).

### Benchmark Results

**Figure 1.**
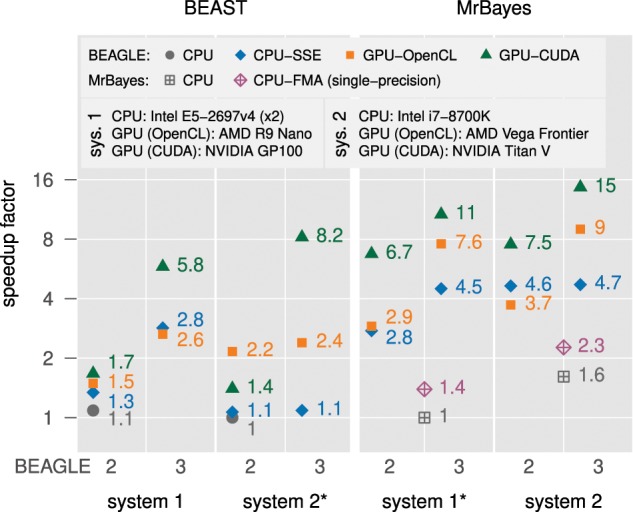
Relative performance gains (fold-speedup) for a challenging highly partitioned nucleotide-model analysis using various combinations of implementations and versions of the BEAGLE library, and hardware resources, with BEAST and with MrBayes. We report fold-speedup on the log-scale, relative to the total run time when using the native double-precision likelihood calculator on the slowest system (denoted with an asterisk) for each program.

Figure 1 shows that for both BEAST and MrBayes, and for all hardware resources and corresponding implementations, total run time for this challenging data set improves when using BEAGLE 3. The biggest improvement we observe is for the NVIDIA Titan V GPU under CUDA on *System 2*, where the speedup over the baseline likelihood calculator went from 1.4-fold to 8.2-fold when using BEAST. This corresponds to a 5.9-fold increase in performance due to the improvements in BEAGLE 3. For this same GPU with MrBayes, we observe an improvement from 7.5-fold to 15-fold, which represents a 2-fold gain from using Version 3 of the library.

For the OpenCL implementation in BEAGLE, running on the AMD R9 and Vega GPUs, we also observe clear performance gains from Version 3 of the library. However, we note that in our experience the current version of the AMD OpenCL platform is less mature than the CUDA platform from NVIDIA, and we have observed inconsistent performance with the AMD solution. This issue is especially notable for the BEAST result on *System 2* under BEAGLE 3, where we expected better than the observed 1.1-fold improvement over the previous version of the library.

Another notable result is the performance improvement for the CPU implementation of BEAGLE when using the multicore processor on *System 1*. For both BEAST and MrBayes, we observe gains on the order of 2-fold when using Version 3 of the library. Despite the fact that GPUs can achieve significantly better performance, CPU performance remains highly relevant. Many systems do not have a high-end GPU or might have compatibility issues with the external frameworks required for GPU computing (i.e., CUDA and OpenCL). The CPU implementation in BEAGLE 3 remains highly portable and provides a reliable, yet well-optimized level of performance.

## Scalability

Phylogenetic analysis problems span a range of sizes with dimensions quantified in numbers of characters (e.g., nucleotides, amino acids, codons) and number of operational taxonomic units (OTUs). Therefore, it is pertinent to know if any specific analysis problem fits within available memory, how it might scale across hardware devices, and what are the expectations for performance for the problem size and type. These issues are explicitly addressed in the following subsections.

### Scaling Memory

Memory usage is a relevant concern when evaluating the suitability of GPU acceleration for a phylogenetic analysis. Typically GPUs have less memory than what is available to CPUs, and the high cost of transferring data between CPU and GPU memory prevents direct use of CPU memory for GPU acceleration. Thus it can be important to consider if the GPU being used has sufficient on-board memory.

**Figure 2.**
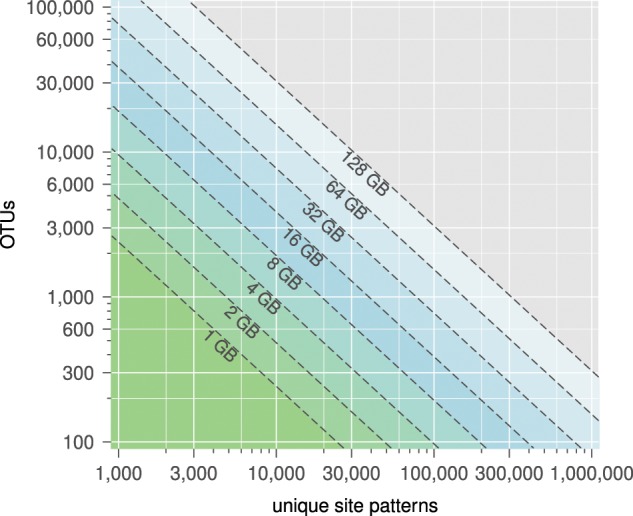
Log–log contour plot depicting BEAGLE-GPU memory usage for BEAST and MrBayes nucleotide-model analyses with four rate categories and double precision floating-point arithmetic, over a range of problem sizes in terms of number of OTUs and of unique site patterns. The amount of memory depicted as values below the dashed isolines convey the upper boundary for the memory size indicated.

BEAGLE memory usage depends on the data type (e.g., nucleotide, codon), evolutionary model characteristics (e.g., number of rate categories), computational precision (i.e., single or double floating-point format), and data set size. Here, we provide some precise estimates of GPU memory requirement for BEAST and MrBayes nucleotide-model analyses with four rate categories and double precision floating-point arithmetic, over a range of problem sizes in terms of number of OTUs and of unique site patterns (Fig. 2).

For perspective, a current model single GPU designed for HPC can have as much as 32 GB of memory (e.g., AMD FirePro S9170, NVIDIA Tesla V100), a single node on the Comet Supercomputer with four NVIDIA Tesla P100 GPUs has $CDATA[$CDATA[$4 \times 16$$ GB for 64 GB of total GPU memory, and single chassis-multiple GPU systems are available currently with up to 512 GB of total GPU memory. We also note that BEAST can distribute data sets across multiple GPUs; thus, each GPU will only require as much memory as necessary for the data subset assigned to it (Fig. 3 shows results for a benchmark that makes use of this feature).

#### Data partitioning

Memory requirements shown in Figure 2 assume an unpartitioned data set. Precise requirements for multiple data subsets depend on many factors, and involve additional memory for independent modeling of each subset, as well as an overhead factor. Compared with previous versions, BEAGLE 3 is more memory efficient and significantly decreases the memory overhead requirement for multiple data subsets. As an example, for the previously described dengue virus data set with 10 partition-defined data subsets (see Performance Evaluation section) BEAGLE 2 requires 7.2 GB, Version 3 requires 3.3 GB. An unpartitioned version of the same data set requires 3.0 GB with BEAGLE 3 and 3.7 GB with Version 2.

### Scaling Computation

BEAGLE has been designed so that a library instance efficiently uses the computing potential of a single hardware resource (i.e., a GPU, or set of CPUs on a single system). In this way, the library makes use of up to tens of cores available in or among CPU(s) or up to thousands of cores available from a GPU within a chassis. Phylogenetic analyses with small and intermediate-sized data sets fit within the memory capacity of a single device (Fig. 2) and can achieve decreased time to results by using a single library instance, when compared with distributed computing approaches. For analyses that benefit from additional computational capacity (from a memory usage or performance standpoint), it is necessary to consider how computation can be distributed and how it scales (over multiple GPUs or over multiple nodes). Here, we explore two approaches to distributing the likelihood computation at the core of phylogenetic analyses.

#### By pattern block

**Figure 5.**
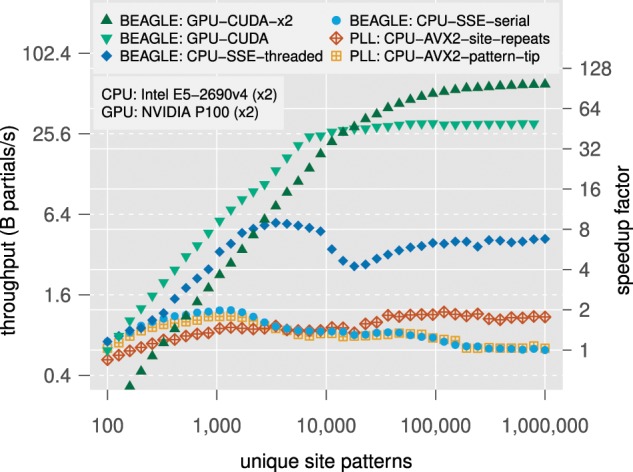
Absolute (throughput in billions of partial likelihood calculations per second) and relative (fold-speedup relative to the slowest performance observed at any number of unique site patterns) performance scaling with problem size for implementations of the BEAGLE library Version 3.1.2 and the Phylogenetics Likelihood library Version 2 on nodes of the Comet Supercomputer available via CIPRES. The data are simulated nucleotide sequences for a tree of 128 OTUs.

Data sets with a sufficiently high number of unique site patterns may saturate the many cores on a GPU or its memory capacity (see Figs. 2 and 5 for nucleotide-model examples). A natural approach to distributing the likelihood calculation is to break up the site patterns into blocks or subsets and to compute these independently. With BEAGLE, pattern blocks can be computed on distinct devices by creating multiple library instances, one for each block. This method is available in BEAST and allows for data sets to be distributed for computation on multiple GPUs.

Figure 3 shows relative performance results for a codon-model analysis with BEAST on a variety of BEAGLE implementations and serves as an example of pattern block scaling. For the benchmarks running on the GPU-CUDA implementation, the required memory exceeded the capacity of a single GPU on either of the two systems tested. Thus, we distributed the computation across two GPUs by splitting the data set into two pattern blocks, each running on a separate library instance (and under a separate thread in BEAST). On both systems, we observe strong performance for the distributed test on two GPUs. On *System 1*, which has $CDATA[$CDATA[$36$$ CPU cores, the dual-GPU speedup over the best-performing CPU implementation is $CDATA[$CDATA[$4.7\times$$. On *System 2*, which has a latest-generation $CDATA[$CDATA[$6$$-core Intel processor, this speedup is $CDATA[$CDATA[$21\times$$. We also highlight the significant performance advantage of the CPU-based OpenCL implementation available in BEAGLE 3 over other CPU approaches. In contrast to traditional implementations, the OpenCL version also parallelizes the character state-count dimension (see Key Improvements section), enabling concurrent computation of the distinct states each sequence character can assume ($CDATA[$CDATA[$61$$ for this codon-based analysis).

#### By Markov chain or run replicate

For Bayesian inference programs that support Metropolis-coupled Markov chain Monte Carlo (Geyer 1991) or multiple runs, another approach to distribute computation is through parallel Markov chains or replicate runs. MrBayes employs this method via Message Passing Interface (MPI) to distribute computation over multiple processes running on a multicore CPU or more widely on a computer cluster (Altekar et al. 2004). This MPI-based approach can be combined with BEAGLE such that each process can use separate library instances and can run on a different device or node, increasing scale in terms of both computation and memory.

**Figure 4.**
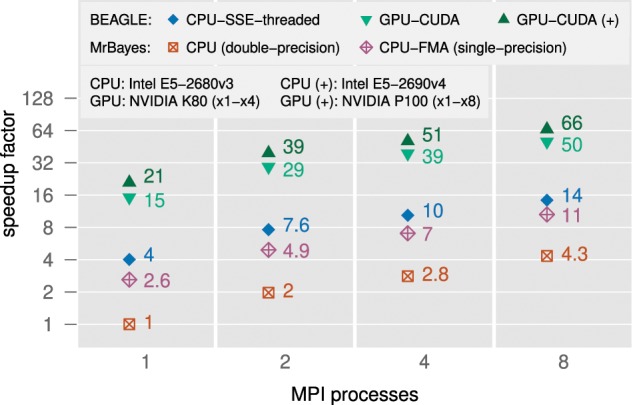
Relative performance gains (fold-speedup) for analysis with MrBayes v3.2.7 and BEAGLE v3.1.2, demonstrating the scalability across a range of MPI processes and hardware devices on nodes of the Comet Supercomputer available via CIPRES. Results denoted with a $CDATA[$CDATA[$(+)$$ were benchmarked on nodes with a newer-specification CPU and GPU (the CPU executed non-BEAGLE code). As reference, we use the double-precision MrBayes calculator, running as a single CPU process. We use the same dengue virus data set as before (see Performance Evaluation section), with an increase in the number of Markov chains to four and replicate runs to two.

To demonstrate the use of the BEAGLE library with MPI for distributing computation, we here provide an example of multi-node benchmarks, performed by Dr. Wayne Pfeiffer of the San Diego Supercomputer Center, on the Comet supercomputer available via CIPRES. Figure 4 shows MPI scalability available with MrBayes v3.2.7 and BEAGLE 3 for Markov chains and run replicates on an increasing number of MPI processes. For the GPU implementation, we contributed improvements to MrBayes so that each process ran on a separate device (an NVIDIA K80 GPU is seen as two devices) and up to $CDATA[$CDATA[$8$$ GPUs were used simultaneously (over two nodes). For the CPU-based implementations, each MrBayes process ran on a separate core on a single node. For all implementations tested, we observe that performance scales similarly as we increase the number of MPI processes from one to eight and that the relative performance advantage of using BEAGLE is retained, independent of the number of processes used.

### Scaling Performance

Relative performance for the different likelihood-calculation implementations available in BEAGLE varies significantly with data set size and evolutionary model employed. In this section, we focus on how performance scales with the number of unique site patterns, the primary dimension of independent likelihood-calculation that is parallelized by all implementations in BEAGLE. Version 3 of the library also parallelizes likelihood computation on the tree toplogy on GPUs (see Key Improvements section) and we have found performance to scale strongly with tree size, resulting in speedups of up to $CDATA[$CDATA[$\sim8\times$$ for trees with over 1000 tips (Ayres and Cummings 2017a).

#### Nucleotide models

We have conducted tests to evaluate how likelihood-calculation performance in BEAGLE scales with the number of unique site patterns for a typical nucleotide-model analysis. For comparison, we also include results using the Phylogenetic Likelihood Library (PLL) (Flouri et al. 2015) Version 2 (commit eda16a6). Currently, there are no phylogenetic analysis programs that can use both BEAGLE and the PLL, and comparing across different programs would involve confounding factors and impacts of the phylogenetic software integrating the library. Thus, performance evaluations were done using a dedicated testing program (*synthetictest*) that generates synthetic data and exercises the core functions of the libraries. This test program is available with the BEAGLE source code and comparison to the PLL can be replicated by setting the with-pll compilation option. Here, the evaluations performed were facilitated because recent versions of the PLL have an API that is similarly modeled to the one in the BEAGLE library. For double-GPU benchmarks, we used recently added functionality in BEAGLE that enables asynchronous API calls to the library, thus allowing concurrent computation on two devices from a single-threaded program. This approach is less efficient than using multithreading to manage multiple library instances (as is done with BEAST) but avoids complexities associated with threading.

The PLL does not support single-precision arithmetic, whereas the BEAGLE library supports both single and double-precision arithmetic, hence all tests used double-precision floating-point arithmetic (single-precision arithmetic is used, often optionally, by some Bayesian analysis programs). The tests also did not perform numerical value rescaling, as there are numerous ways such rescaling procedures can be effected, and this is a design feature that differs among phylogenetics programs. Rescaling is often necessary as the likelihood values for many data sets lead to arithmetic underflow, a state where floating-point numbers are smaller than the limit of what is representable by the processor, and hence have to be rescaled to maintain necessary precision. With BEAGLE we typically observe an $CDATA[$CDATA[$8\%$$ cost on overall performance when using numerical rescaling factors for likelihood evaluation.

Dr. Wayne Pfeiffer of the San Diego Supercomputer Center independently executed the benchmark tests on the Comet supercomputer, which is among the resources backing the CIPRES Science Gateway (Miller et al. 2010), and thus available to the broader systematics community. He employed a Comet node with dual Intel Xeon E5-2690v4 processors and four NVIDIA Tesla P100 GPUs (only two GPUs were used in the tests shown). We make the benchmark scripts used available in the BEAGLE source code repository (see Availability section).

The tests first simulated nucleotide data for a tree of $CDATA[$CDATA[$128$$ OTUs using an evolutionary model with $CDATA[$CDATA[$4$$ (arbitrary value) rate categories and then computed the likelihood for $CDATA[$CDATA[$10$$ variations of the tree and model parameters (specifically, the tests varied topology, branch lengths, category rates, category weights, and pattern weights). The tests did not include branch-length optimization, a ML specific operation. BEAGLE can compute first and second branch-length derivatives (at a performance cost of $CDATA[$CDATA[$\sim4\%$$ for a data set with 128 OTUs running on the GPU implementation); however, the numerical optimization procedure to propose new branch-lengths needs to be carried out by the client program and thus would not be a measure of library performance. The tests replicated each run $CDATA[$CDATA[$10$$ times with different starting random seeds and reported the mean performance across the replicates. The number of unique site patterns ranged from $CDATA[$CDATA[$1 \times 10^{2}$$ to $CDATA[$CDATA[$1 \times 10^{6}$$. Note that for most data sets the number of unique sites is substantially less than the aligned sequence length, and hence these values correspond to larger data sets of actual nucleotide sequences.

We collected performance results for the two best-performing implementations of the PLL and for all implementations of BEAGLE that are appropriate for the CIPRES hardware. These comprised the following implementations: the PLL CPU with AVX2; the PLL CPU with AVX2, and using a novel algorithm to reduce computation associated with repeated site states (Kobert et al. 2017); the BEAGLE library CPU with SSE; the BEAGLE library CPU with SSE and threading; and the BEAGLE library GPU with CUDA, a programming framework for computing on NVIDIA GPUs. We note that the PLL Version 2 does not include multi-core implementations (e.g., via multithreading or MPI).

We measured both absolute performance, defined as throughput in units of billions of partial likelihood calculations per second, and relative performance, defined as fold-speedup relative to the slowest performance observed for the BEAGLE library CPU with SSE at any number of unique site patterns. The comparative performance results for the BEAGLE library and the PLL are presented in Figure 5.

For problem sizes greater than $CDATA[$CDATA[$10^2$$ site patterns, best performance is achieved with the BEAGLE CUDA implementation running on one or two NVIDIA P100 GPUs. With a single GPU, performance increases until $CDATA[$CDATA[$10^4$$ patterns, where the hardware reaches a saturation point at $CDATA[$CDATA[$\sim25.6$$ B partial-likelihood operations per second (representing a speedup of $CDATA[$CDATA[$\sim32\times$$ over single-threaded CPU implementations). When using two GPUs (in asynchronous mode with our single-threaded test program), this saturation point is shifted further towards larger problem sizes. On the CPU, we find the BEAGLE-SSE implementation with threading achieves best performance at any problem size, with the relative gain increasing with problem size. For single-threaded CPU implementations, BEAGLE-SSE is fastest up to $CDATA[$CDATA[$10^3$$ patterns, and the PLL AVX-2 site-repeats implementation performs best for problems above $CDATA[$CDATA[$10^3$$ patterns (up to a peak of $CDATA[$CDATA[$\sim2\times$$ over other single-threaded implementations, for very large problems).

In order to more comprehensively assess the performance of the PLL site-repeats implementation, we repeat the comparative benchmarks described in this section on a sample of empirical multiple sequence alignments with corresponding parsimony trees, each with a high proportion of repeated sites ($CDATA[$CDATA[$>90\%$$) (Kobert et al. 2017). Table 1 shows fold-speedups for each of these data sets, defined relative to the performance observed for BEAGLE CPU with SSE. We find that the BEAGLE GPU implementation is fastest at any problem size, with speedups of up to $CDATA[$CDATA[$37\times$$ over the reference serial implementation. On the CPU, the multithreaded approach in BEAGLE is fastest for all but the smallest data set (measured by number of unique site patterns). If we only consider single-threaded CPU approaches, the PLL solution using site-repeats performs best, with speedups of up to $CDATA[$CDATA[$3.1\times$$ over the single-threaded BEAGLE implementation.

**Table 1.** Relative performance for implementations of BEAGLE Version 3.1.2 and the PLL Version 2 for a sample of empirical data sets with an increasing number of unique site patterns and a high percentage of repeated sites

	Unique site patterns	348	3224	7418	19,437
Speedup vs. BEAGLE SSE	OTUs (sequences)	354	59	404	128
	Repeated sites (%)^a^	94.65	92.04	96.49	91.78
	PLL AVX2-pattern-tip	0.88	0.92	0.96	0.98
	PLL AVX2-site-repeats	2.28	1.85	3.10	2.31
	BEAGLE SSE-threaded	1.63	5.72	5.24	5.23
	BEAGLE GPU-CUDA	3.36	21.82	35.49	37.06

*Note*: Benchmarks performed on an Intel Xeon E5-2697v4 CPU and on an NVIDIA GP100 GPU. All implementations use a single CPU thread except for the BEAGLE SSE-threaded implementation that uses up to eight threads. Data sets are a sample of those used in Kobert et al. (2017). We generated trees by running a parsimony tree search with Parsimonator (Stamatakis 2014) with arbitrary rooting.

^a^Repeated sites denote the number of sites over all nodes that are repeats of another site at the same node, and thus depends on the tree topology, the selected root, and the data set. As an approximate reference, we reproduce the percentage of repeated sites for each data set that Kobert et al. (2017) report, where they used an independently generated parsimony tree and indeterminate random rooting.

#### Other models

For amino acid and codon-based models, we observe GPU performance to be less sensitive to the number of unique site patterns (Ayres and Cummings 2017b; Baele et al. 2018). This is due to the better parallelization opportunity afforded by the increased number of states that can be encoded by an amino acid or codon. The higher state count of these data types compared to nucleotide data increases the ratio of computation to data transfer, resulting in increased GPU performance (Fig. 3).

## Usability

Since the first release of BEAGLE, we have received occasional feedback from researchers performing analyses where use of the library did not meet performance expectations or that it failed to work at all. In general, such issues occur due to the use of under-powered GPUs, such as those found on notebook computers, or due to incorrect or missing installation of the necessary CUDA or OpenCL frameworks. For guidance, users can refer to the online documentation (see Availability section) for the library and to the specific instructions for each application.

### Automatic Resource Selection

Beyond the issues described above, which relate to the configuration of the system being used, the characteristics of the data set and evolutionary model employed can also have a significant impact on performance or even preclude the use of a GPU (see Scalability section). Choosing the best-performing implementation across various hardware devices has previously required the user to evaluate available resources (e.g., CPU, GPU) with analysis parameters and data set characteristics specific to the problem at hand. To eliminate this additional level of complexity for the user, we have extended the BEAGLE library API so that it now supports benchmarking that provides a ranking of available hardware resource and implementation combinations for the analysis parameters to be used in the target analysis. Furthermore, we have added this automatic resource selection feature to BEAST (Version 1.10.5) and MrBayes (Version 3.2.7).

### Support in Phylogenetic Software Packages

The current extent and status of BEAGLE integration with different phylogenetic software packages is varied. The latest versions of Bayesian-inference programs BEAST (Suchard et al. 2018) (v1.10.5) and MrBayes (Ronquist et al. 2012) (v3.2.7) feature the most complete support for the library and for the improvements here described. BEAST2 (Bouckaert et al. 2014), an independent project to BEAST, also features extensive support for the library but does not make use of the latest advances such as increased parallelism for nucleotide-model analyses on GPUs. Specific advice on how to use BEAGLE with these programs is available through their documentation (online and at runtime).

The BEAGLE API and library implementations (CPU and GPU based) also provide support for ML programs via methods specific to this approach (such as branch-length derivative calculation). These methods have been available since the first release of the library and significant speedups have been observed with a development-version of GARLI (Zwickl 2006; Ayres et al. 2012). At present, support for BEAGLE in ML-based programs remains experimental or in-development. Development branches supporting the library are publicly available in the GARLI and PHYML (Guindon et al. 2010) source code repositories, and work is in-progress for PAUP* (Swofford 2003). The considerable performance benefits of using the BEAGLE library on even desktop computers provide an incentive for continued development of these projects and for other software developers to explore doing so.

## Documentation for Developers

Creating software that uses any library can be challenging without sufficient documentation, and the complexity of both the BEAGLE library itself and the phylogenetic applications for which it is designed can make entry difficult for beginning users. An online tutorial (see Availability section) shows how to use the BEAGLE library to greatly simplify the efficient calculation of the likelihood of sequences on a phylogenetic tree. The tutorial explains how to 1) set up a project that links the BEAGLE library under two common freely-available integrated development environments, 2) construct C++ classes that manipulate trees and process data, and 3) use the BEAGLE library to calculate the likelihood under the GTR+G (Tavaré 1986; Yang 1996) substitution model as well as arbitrary rate matrices. This tutorial serves to augment the BEAGLE library API documentation with an extensive example application in C++. The same principles can also be applied to write applications in other languages for which BEAGLE includes wrappers (currently Java, and Python with partial functionality).

## Conclusion

The BEAGLE library addresses a common bottleneck across phylogenetic inference programs by accelerating likelihood computation. Among other improvements, Version 3 of the library includes additional parallel computing advances and combines CUDA, OpenCL, and native CPU-threading implementations in a single codebase to address a wider-range of hardware resources. Additionally, increased concurrency of computation for large trees and partitioned data sets allows a wider range of phylogenetic inferences to benefit from GPU acceleration. These advances serve as an important step in combining the capabilities of increasingly parallel hardware with the demands of progressively more sophisticated phylogenetic analyses.

## Availability

The BEAGLE library is free, open-source software licensed under the Lesser General Public License (GPL). The source code, benchmark files, documentation, as well as binary installers for macOS and Windows, are available at https://beagle-dev.github.io. An online tutorial for application developers is available at https://stromtutorial.github.io.
